# Extraction and Structural Characterization of Four Grape Polysaccharides and Their Protective Effects in Alcohol-Induced Gastric Mucosal Injury

**DOI:** 10.3390/foods13213500

**Published:** 2024-10-31

**Authors:** Jian Shao, Jizhen Li, Yonghui Zhao, Rong Huang, Aixin Guo, Lijuan Hou, Xiangpeng Leng, Qiu Li

**Affiliations:** 1 National Joint Local Engineering Laboratory of Agricultural Bio-Pharmaceutical Laboratory, College of Chemistry and Pharmaceutical Sciences, Qingdao Agricultural University, Qingdao 266109, China; 15621090551@126.com (J.S.); ljz1003409288@163.com (J.L.); e_huangrong@163.com (R.H.); gax1201@139.com (A.G.); lengpeng2008@163.com (X.L.); 2 Beijing Shengtaier Technology Co., Ltd., Beijing 100083, China; 3 Qingdao Central Hospital, University of Health and Rehabilitation Sciences, Qingdao 266000, China; longqin5768@163.com; 4 Weihai Academy of Agricultural Sciences, Weihai 264299, China; houlijuan-2007@163.com

**Keywords:** grape polysaccharides, structural characterization, gastric mucosal protection

## Abstract

Grapes, recognized as a nutritionally rich fruit, have been found through extensive research to contain various bioactive components. However, the roles of polysaccharides and their bioactive properties remain unclear. Based on this, in our research, four different grape polysaccharides were obtained using an enzymatic-assisted extraction method. We investigated and compared their physicochemical properties, antioxidant activities, and protective effects on gastric mucosa in mice. The results indicated that the monosaccharide compositions of these specific grape polysaccharides were similar; however, their molar ratios, molecular weights, and morphological characteristics varied. The results of radical scavenging tests revealed that red-fleshed grape polysaccharide (RFP) exhibited superior antioxidant properties. In vivo assessments demonstrated that RFP protects against gastric mucosal injury in mice by inhibiting inflammation and radical generation. Therefore, the polysaccharide from red-fleshed grape holds potential application value in the pharmaceutical and food industries.

## 1. Introduction

Polysaccharides are a class of macromolecular polymers consisting of the linkage of monosaccharide molecules through glycosidic bonds [1]. They are widely distributed in nature [2], including within plants [3], animals [4], and microorganisms [5]. Due to their unique physicochemical properties and biological activities, polysaccharides exhibit broad application potential in various fields [6]. In the biomedical sciences, polysaccharides are being studied as drug delivery systems [7], scaffold materials [8], and wound dressings [9]. Their applications in the food industry are also extensive, serving as thickeners [10], gelling agents [11], and emulsifiers [12] to improve the texture, stability, and mouthfeel of food products. The rheological properties of polysaccharides, such as thickening, gelling, and emulsifying, are closely related to their molecular structure. Additionally, polysaccharides possess various biological activities, including immunomodulation [13], antiviral effects [14], antioxidant properties [15], blood glucose reduction, and gastrointestinal protection [16]. In summary, the application fields of polysaccharides are vast, demonstrating their unique value across biomedical and food industries.

In recent years, gastrointestinal health has attracted increasing attention, particularly regarding the protection against gastric mucosal injury, which has become a research hotspot. Currently, the treatment for gastric mucosal damage in clinical practice primarily involves the use of chemically synthesized drugs. These medications include proton pump inhibitors [17], antibiotics, antihistamines, and antacids [18]. Although these drugs can provide temporary symptom relief for patients, they often do not offer a fundamental solution and may be associated with varying side effects with long-term use [1]. Therefore, researchers have begun to actively explore the potential of natural macromolecular compounds, particularly polysaccharides, to discover new molecules that can safely and effectively protect against gastric mucosal injury. For example, it has been reported that cardamom polysaccharides alleviate the adverse effects of ethanol-induced acute gastric mucosal injury in rats by enhancing antioxidant capacity and immunity while reducing apoptosis [19,20]. Additionally, *Dendrobium officinale* polysaccharides can protect the gastric mucosa by inhibiting the expression of genes and proteins in the MAPK pathway, thereby lowering the levels of inflammatory factors [21].

Grapes are one of the most popular fruit crops, offering significant nutritional and health benefits. It has been reported that grapes contain a series of antioxidants [22], including polyphenols, proanthocyanidins, and resveratrol. Polysaccharides are the primary macromolecular compounds found in grapes, wine, and grape juice [23]. Grape polysaccharides are mainly derived from the cell walls of grape flesh and skins, and are a major component of dietary fiber [24]. Grape polysaccharides generally exhibit antioxidant activity, being capable of scavenging free radicals and reducing oxidative stress-induced cellular damage. Additionally, they can protect cells from oxidative damage by enhancing the effects of antioxidant enzymes [25].

Based on this, we considered investigating whether grape polysaccharides have unique advantages in protecting against gastric mucosal injury. We selected four grape varieties for our study, including *Vitis davidii* (Rom. Caill.) Foëx.(VF), *Vitis rotundifolia* Michx.(VM), red-fleshed grape (RF), and Marselan grape (ML). Firstly, we obtained polysaccharides from these four grape varieties through enzyme-assisted extraction, characterizing their physicochemical properties and structures. We further assessed their antioxidant activity and established a mouse model of alcoholic acute gastric mucosal injury to evaluate the protective effects of the selected polysaccharide on the gastric mucosa. We innovatively obtained polysaccharides from four distinct grape varieties for the first time, conducting research on their specific bioactivities, with a particular focus on investigating their protective effects against alcohol-induced gastric mucosal injury. Our research may provide insights for the development of new therapeutic foods based on this novel polysaccharide.

## 2. Materials and Methods

### 2.1. Grapes and Physiological Parameter Measurement

Four grape varieties were purchased from Qingdao, Shandong, China: *Vitis davidii* (Roman. du Caill.) Foex (VF), *Vitis rotundifolia* Michx (VM), red-fleshed (RF), and Marselan (ML). The grape clusters were harvested at the ripening stage of each grape variety. Thirty berries were randomly selected for physiological analysis, including individual berry weight, longitudinal and total soluble solids, transverse diameter, and titratable acidity.

### 2.2. The Extraction of Polysaccharides Assisted by Enzyme

We prepared polysaccharides from four types of grapes using an enzyme-assisted extraction method. Fresh grapes (VF, VM, RF, and ML) were weighed at 100 g each, crushed, and dispersed in distilled water. The pH of the suspension was adjusted to 5.0 by adding pectinase. The enzymatic hydrolysis time ranged from 20 to 100 min, the temperature was maintained between 45 and 65 °C, and the liquid–solid ratio was between 2 and 6 mL/g. The suspension was then rapidly heated to 95 °C for 20 min to inactivate the enzymes. After centrifugation at 25 °C (4000 rpm for 30 min), the supernatant was collected and concentrated, and ethanol was added to achieve a final concentration of 80% (*v*/*v*). The solution was stored overnight at 4 °C. The supernatant was discarded, and the precipitate was collected. The precipitate was dissolved in deionized water, and an equal volume of petroleum ether was added for defatting. Chloroform and n-butanol (4:1 *v*/*v* ratio) were added to the treated polysaccharide solution, which was then vigorously shaken for 20 min and centrifuged at 4000 rpm for 5 min to remove proteins. This process was repeated several times until a protein-free solution of grape polysaccharides was obtained.

The proteoglycan-free polysaccharide solution was decolorized and pigments were removed using an AB-8 resin column. The column dimensions were 20 mm in outer diameter and 30 mm in height. Following decolorization, the polysaccharide solution was dialyzed in a dialysis bag for 48 h to remove a series of molecules, resulting in a purified grape polysaccharide solution. After freeze-drying, polysaccharides from four types of grapes (VF, VM, RF, and ML) were obtained and designated as VFP, VMP, RFP, and MLP, respectively.

### 2.3. The Box–Behnken Design and Analysis

Based on previous research findings, three critical factors have been identified to significantly influence polysaccharide yield: enzymolysis time, water bath temperature, and liquid–solid ratio. We employed single-factor experimental design as our research strategy. In this design, we systematically investigated these three key factors. Specifically, in each experiment, only one factor was varied within a predetermined range while maintaining the other two factors constant. This approach enabled us to precisely observe the direct effect of individual factor variations on polysaccharide yield while eliminating interference from other variables. Subsequently, a response surface analysis was performed using the Box–Behnken design (BBD), conducting 17 trials at three levels for the aforementioned three factors (33). The range of values for the independent variables, their levels, and the outcomes of the entire design, which comprised 17 randomly assigned experimental points, are presented in Table 1. Data processing and analysis were conducted using Design-Expert 13.0 software.

### 2.4. Characterization of Polysaccharides from Grapes

#### 2.4.1. Determination of Molecular Weights

The molecular weight (Mw) of grape polysaccharides was determined by high-performance size exclusion chromatography (HPSEC) on an HPLC system (Agilent Corporation, Santa Clara, CA, USA) with a TSK-Gel G4000 SW_XL_ column (7.8 mm × 300 mm, Tosoh Biosep, Tokyo, Japan) and an RID refractive index detector. Grape polysaccharide extracts (5 mg) were dissolved in 1.0 mL of distilled water. The chromatographic conditions were as follows: injection volume, 5 µL; temperature, 35 °C; mobile phase, ultrapure water; and flow rate, 0.7 mL/min. Dextran standards with varying molecular weights (10, 40, 70, 200, 500, 1000, and 5300 kDa) were used to generate a calibration curve for estimating the molecular weights of the four grape polysaccharides.

#### 2.4.2. The Monosaccharides Ratio

The monosaccharide ratio of four grape polysaccharides was measured by ion chromatography as previously described. Approximately 5 mg of the sample was hydrolyzed with 2 M trifluoroacetic acid at 121 °C for 2 h in a sealed tube. The sample was then dried with nitrogen. The mixture was washed with methanol, blow-dried, and this methanol washing process was repeated three times. The residue was redissolved in deionized water and filtered through a 0.22 µm microporous filtration membrane for subsequent use. The sample was analyzed by high-performance anion-exchange chromatography (HPAEC) using a CarboPac PA-20 anion-exchange column (3 × 150 mm; Dionex) and a pulsed amperometric detector. Monosaccharide standard solutions, containing rhamnose (Rha), arabinose (Ara), galactose (Gal), glucose (Glc), xylose (Xyl), mannose (Man), fucose (Fuc), fructose (Fru), glucuronic acid (GlcA), galacturonic acid (GalA), and mannuronic acid (ManA), were analyzed as described above.

#### 2.4.3. FT-IR Assay

The functional groups of four grapes polysaccharides were analyzed using Fourier-transform infrared spectroscopy (FT-IR). The FT-IR spectrometer utilized (Spectrun100, Thermo Fisher Scientific, Waltham, MA, USA) measured the wavenumbers from 400 to 4000 cm^−1^.

#### 2.4.4. SEM Present

The morphological characteristics of the polysaccharides were detected by the Hitachi S-3400N-II SEM (Tokyo, Japan) instrument. Polysaccharides and gold powder were placed on a fixture to conduct electricity to conduct electricity. The microstructure of the purified fractions was obtained by magnifying the samples at 500× (the voltage of 2 KV, 3.0 nm spot size, 10 µm objective aperture, and 8 mm working distance).

### 2.5. Antioxidant Effects of the Polysaccharides

#### 2.5.1. DPPH Radical Scavenging Activity Measurement

Aqueous solutions of grape polysaccharide at various concentrations were prepared, with sample concentrations being 0.625, 1.25, 2.5, 5, and 10 mg/mL, respectively. DPPH solution was prepared in anhydrous ethanol, shaken, and incubated in the dark at room temperature for 30 min. Using anhydrous ethanol as a blank, we measured the absorbance (A1) at 517 nm. For the blank group, we replaced the sample solution with 3 mL of anhydrous ethanol, processed as above, and measured the absorbance (A0) at 517 nm. For the control group, we replaced the DPPH solution with 1 mL of anhydrous ethanol, processed as above, and measured the absorbance (A2) at 517 nm. Vc (ascorbic acid) was used as a positive control. The test was repeated 3 times in each group, and the average value was calculated. The scavenging rate of hydroxyl radicals was calculated according to the following formula:

DPPH scavenging activity (%) = [1 − (A_1_ − A_2_)/A_0_] × 100%,

A_0_—absorbance without sample; A_1_—absorbance of the added sample; A_2_—absorbance of sample solution with ethanol.

#### 2.5.2. The Measurement of ABTS Radical Scavenging Activity

The ABTS radical scavenging assay was performed using previously reported methods with some modifications [26]. A 0.2 mL sample of polysaccharide solution with different concentrations (0.625, 1.25, 2.5, 5, 10 mg/mL) was mixed with ABTS solution and incubated at room temperature for 6 min. With Vc as positive control, the absorbance was measured at 734 nm. The ABTS scavenging activity was calculated using the following formula:

ABTS scavenging activity (%) = (A_0_− A_1_ )/A_0_ × 100%

A_0_—absorbance without sample;

A_1_—absorbance of the added sample.

#### 2.5.3. The Measurement of Hydroxyl Radical Scavenging Activity

The hydroxyl radical (OH^−^) scavenging effects were measured according to the previous report. A 1 mL sample solution with different concentrations (0.625, 1.25, 2.5, 5 and 10 mg/mL) was put into a plug test tube, and 1 mmol/L ferrous sulfate solution (FeSO_4_), 9 mmol/L hydrogen peroxide solution (H_2_O_2_), and 3 mmol/L salicylic acid–ethanol solution were quickly added. The mixture was evenly mixed and the reaction was carried out at room temperature for 30 min in the dark. The absorbance value of the solution to be measured at 510 nm was determined, and the above experiment was repeated 3 times. The Vc at the same concentration was used as a positive reference to calculate the mean value. The scavenging rate was calculated as follows:

OH^−^ scavenging activity (%) = (A_0_ − A_1_)/A_0_ × 100%

A_0_—absorbance without sample;

A_1_—absorbance of the added sample.

### 2.6. Animal Experiment

#### 2.6.1. Animals

Male SD rats (weight 180 ± 20 g) were purchased from Jinan Pengyue Experimental Animal Breeding Co., Ltd., Jinan, China (Certificate No. SCXK (Lu) 2022-0006) and were approved by the Ethics Committee of Qingdao Agricultural University (IACUC-232015-2). Rats were maintained under standard laboratory conditions with a temperature of 25.0 ± 0.5 °C, a relative humidity of 50 ± 5%, and a 12 h light–dark cycle. Before the experiment, the rats were fed and watered freely and adapted to the experimental environment for 1 week.

#### 2.6.2. Experimental Design

An ethanol-induced gastric mucosa injury model was used to evaluate the preventive effect of RFP. The rats were randomly allocated to control and treatment groups using randomization. The method employed to generate the randomization sequence was the random number table technique. The rats were randomly divided into 4 groups with 5 in each group: control group (control), model group (model), positive group (OMLS) and experimental group (RFP). Omeprazole has a significant protective effect on gastric mucosal injury and is a clinical drug for the treatment of gastric mucosal injury. The control group and model group were given gavage with water (20 mg/kg/d), the positive group was given gavage with omeprazole (20 mg/kg/d), and the experimental group was given gavage with RFP (70 mg/kg/d) for 7 days. After the last gavage, the rats were fasted for 12 h. Except for the control group, the other groups were given absolute ethanol (5 mg/kg) by gavage. One hour later, all rats were sacrificed under anesthesia, then eyeball blood and gastric tissue were collected for examination.

#### 2.6.3. Stomach Tissue Sample for Examination

Following euthanization, an incision was made to access the peritoneal cavity. The stomach was isolated by ligating both the cardiac and pyloric sphincters before being excised. Subsequently, a longitudinal incision was performed along the greater curvature, followed by physiological saline lavage. Macroscopic assessment of mucosal damage was conducted through visual inspection and photographic documentation. The examination protocol involved meticulous observation of ulcerative lesions in the mucosal layer, with precise measurements of ulcer dimensions (length and width) obtained using precision calipers. The severity of mucosal damage was quantified according to the following assessment criteria (Table 2). The gastric mucosal injury index was calculated using the following formula: gastric mucosal injury index = score of bleeding points + score of erosive length + score of erosive width × 2. Finally, part of the gastric tissue was fixed in a 4% paraformaldehyde solution and used for histopathological analysis.

#### 2.6.4. Histological Analysis

Hematoxylin–eosin (HE) staining was used for histopathological observation of gastric mucosa. Paraffin tissue sections (4 μm) were made from the most severe ulcer sites. The sections were stained with hematoxylin and eosin and then examined under a light microscope. The score of gastric mucosal lesions was calculated by the instruction of INHAND (International Harmonization of Nomenclature and Diagnostic Criteria for Lesions in Rats and Mice): necrosis, hemorrhage, connective tissue hyperplasia, edema, and inflammatory cell infiltration were scored separately and summed as the final score. No exception is 0 points; just exceeded the normal range for 1 point; lesions can be observed, but not serious (2 points); the lesion is obvious and likely to be more serious (3 points); very severe lesions were 4 points.

#### 2.6.5. The Biological Activity Test

Various physiological and biological indicators in mouse serum were measured by the kits. According to the manufacturer’s instructions, the secretion of tumor necrosis factor-α (TNF-α), interleukin-1β (IL-1β), interleukin 4 (IL-4), gastrin-17 (Gas-17), pepsinogen I (PG I), pepsinogen Ⅱ (PGⅡ), superoxide dismutase (SOD), glutathione (GSH), malondialdehyde (MDA), and myeloperoxidase (MPO) were detected by the ELISA kits (Jiangsu Meimian, China).

### 2.7. Data Analysis

Statistical analyses were presented with values denoted as means accompanied by their standard deviations (SD). Statistical significance was indicated at two levels: single asterisk (*) for *p* < 0.05 and double asterisk (**) for *p* < 0.01. Data processing was conducted using SPSS software package (version 21.0), while graphical representations were generated through the Origin 2018 platform. The experimental data underwent comprehensive analysis employing Design Expert 13.0 (Stat-Ease Inc., Minneapolis, MN, USA), which facilitated the generation of perturbation plots, execution of regression analyses, and construction of three-dimensional response surfaces.

## 3. Results and Discussion

### 3.1. Assay of Physiological Parameters

**Figure 1:**
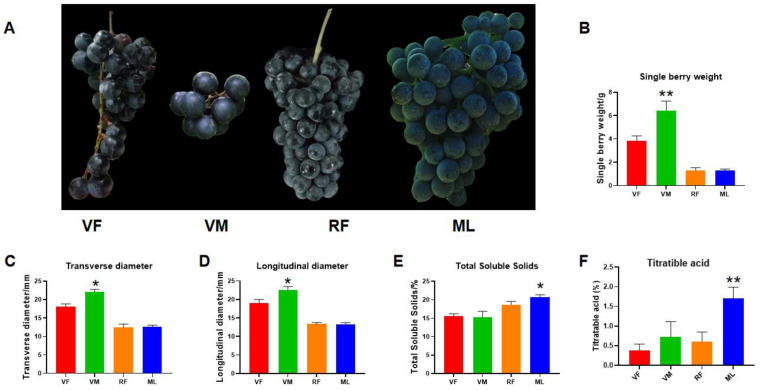
Berry characteristics of four grape varieties. (**A**) Phenotype characteristics of four table grape varieties. (**B**–**F**) Several basic physiological indexes, including single berry weight, transverse diameter, longitudinal diameter, total soluble solids, and titratable acid were measured. Data are shown as the means ± standard deviation (n = 30), VM versus ML, * *p* < 0.05, ** *p* < 0.01.

From the physiological parameters of the four grapes (Figure 1A–D), VM displayed the largest berry weight, transverse diameter, and longitudinal diameter. Total soluble solids (TSS) is a collective term for all the compounds in grape berries that can be dissolved in water. The main components of these compounds are sugar (such as glucose, fructose, etc.) and acid (such as malic acid, tartaric acid, etc.). It also confirms grapes with high TSS content are more intense in flavor and fuller in taste [27]. Titratable acidity represents the total amount of acid substances in grapes, mainly malic acid and tartaric acid, which is one of the key factors affecting the taste and quality of wine. As can be seen from Figure 1E,F, the results represent the total soluble solids (TSS) and titratable acidity (TA) of grapes, respectively. Total soluble solids encompass all water-soluble compounds present in grape berries, predominantly consisting of sugars (such as glucose and fructose). Titratable acidity refers to the total amount of acidic substances in grapes, primarily malic acid and tartaric acid. The graphs indicate that ML exhibited the highest levels of both TSS and TA.

### 3.2. The Optimizing of Parameters on the Yield of Grape Polysaccharides

#### 3.2.1. Single Factor Experimental Analysis

**Figure 2:**
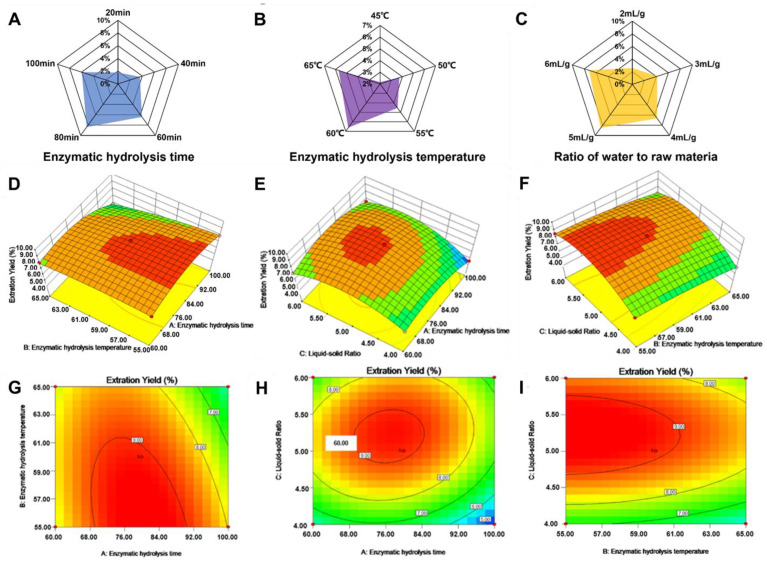
Interactions of different operation parameters on the extraction yield of grape polysaccharides. The effect of different enzymatic hydrolysis times (**A**), enzymatic hydrolysis temperature (**B**), and ratio of water to raw material (**C**) on the yield of grape polysaccharides in single-factor experiments. The 3D and 2D plots showing the effects of different extraction parameters on extraction yield of grape polysaccharides, respectively (**D**–**I**).

Based on the single factor experiment, three critical parameters—enzymolysis time, temperature and liquid–solid ratio—were selected to investigate the effects of polysaccharide yield. Initially, the effect of different enzymolysis time on polysaccharide yield was investigated at 55 °C and 4 mL/g. When the enzymolysis time increased from 60 min to 80 min, the yield of grape polysaccharide rapidly increased from 6.21% to 8.17%, and when the enzymolysis time increased to 100 min, the yield of grape polysaccharide gradually decreased to 6.05% (Figure 2A). This is attributed to the fact that, in the initial phase, the extraction rate of polysaccharides increases progressively with the prolongation of the enzyme hydrolysis time. Nevertheless, an excessively long enzymatic hydrolysis time may lead to partial degradation of polysaccharides under the action of enzymes, thus reducing the extraction rate [28].

Similarly, under the conditions of enzymolysis time of 60 min and liquid–solid ratio of 4 mL/g, the polysaccharide yield continued to increase as the enzymolysis temperature was raised from 45 °C to 60 °C, and then decreased when the enzymolysis temperature was further increased to 65 °C (Figure 2B). Within the optimal temperature range, the activity of enzymes is higher, which can accelerate the decomposition and release of polysaccharide, so as to improve the extraction rate of polysaccharide. However, if the temperature is too high, the enzyme may be denatured and deactivated, resulting in the prevention of enzymatic hydrolysis, thus reducing the extraction rate of polysaccharide. When the enzymolysis temperature was 55 °C and the enzymolysis time was 60 min, the polysaccharide yield continued to increase when the liquid–solid ratio was increased from 2 mL/g to 5 mL/g, and the polysaccharide yield decreased when the liquid–solid ratio was increased by 6 mL/g (Figure 2C). It can be seen that a suitable liquid–solid ratio was conducive to the diffusion of polysaccharide from the sample to the solvent, and the extraction rate increased. However, an excessive increase of liquid–solid ratio decreased the yield of polysaccharide.

#### 3.2.2. Response Surface Analysis

The response surface method was employed to evaluate the extraction efficiency of polysaccharides and confirm the optimal process. The extraction rate and estimated response Y of grape polysaccharides can be modeled by the following second-order polynomial equation: Y = 9.10 − 0.53 A − 0.57 B + 0.70 C − 0.50 AB + 0.34 AC − 0.02 BC − 1.39 A^2^ − 0.27B^2^ − 1.61C^2^, where Y represents the extraction yield of grape polysaccharides, and A, B, and C, respectively, represent the enzymatic hydrolysis temperature, enzymatic hydrolysis time, and liquid–solid ratio.

We summarized the ANOVA analysis of the response surface quadratic model in Table 3. A high F-value (296.64) and a low *p*-value (*p* < 0.0001) indicated that the regression model was highly significant. The goodness of fit of the regression model was estimated by the coefficient of determination (R^2^) and the adjusted R^2^. R^2^ (0.9947) and adjusted R^2^ (0.9940) showed a very high correlation between actual and predicted values. In addition, the coefficient of variation (CV) of the method was very low (1.43%), indicating that the method had a high accuracy.

A two-dimensional (2D) contour plot and three-dimensional (3D) response surface plot were used to represent the interaction between the independent variables (Figure 2D–I). The closer the contour map is to a complete circle or oval, the greater the interaction between the two. Overall, grape yield was sensitive to small changes in liquid–solid ratio, which was consistent with the results of statistical analysis. According to the test results, the optimal technological conditions for polysaccharide extraction were as follows: enzymatic hydrolysis time was 74.6 min; enzymatic hydrolysis temperature 56.4 °C; liquid–solid ratio 5.1 mL/g. The maximum yield of grape polysaccharide predicted by this model was 9.35%.

### 3.3. Structural Characterization of Polysaccharides

#### 3.3.1. Molecular Weight and Monosaccharides Composition

**Figure 3:**
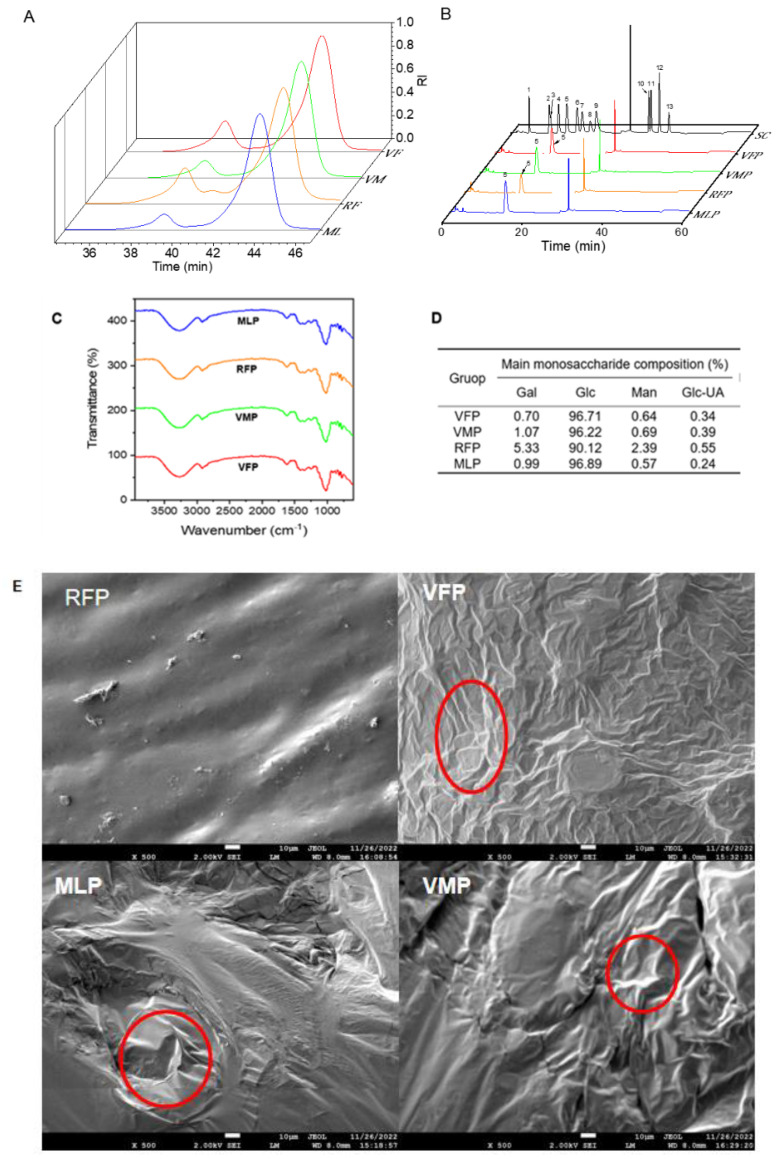
The characterization of the different polysaccharides. (**A**) HPSEC of molecular weight for the polysaccharides. (**B**) The monosaccharide constituents of the polysaccharides. The standards were Fuc, Ara, Rha, Gal, Glc, Xyl, Man, Fru, Rib, GalA, GulA, GlcA, and ManA, SC was standard curve. (**C**) FT-IR of the polysaccharides. (**D**) The molecular weight and main monosaccharide composition ratio of grape polysaccharides. (**E**) SEM images of four polysaccharides (the red-circled area indicates the region of differentiation).

Polysaccharides from natural plants have complex structures and various types. As an important characteristic parameter of polysaccharides, relative molecular weight has an important impact on their biological activities and applications. As shown in Figure 3A, all the polysaccharides showed different peaks, indicating that these grape polysaccharides had different molecular weights. The results showed that RFP had the highest molecular weight (78.5 KDa), followed by VFP (53.6 KDa), MLP (66.7 KDa), and VMP (46.2 kDa).

The monosaccharides are the basic units controlling the unique structure, characterization, and biological activity of polysaccharides, and the diversity of monosaccharide composition may lead to individual differences in biological activity. The results in Figure 3B show 13 monosaccharide were determined by HPLC, and it was found that the main component of these four grape polysaccharides was Glc (more than 90%), followed by Gal, Man, and GlC-UA. The contents of Gal (5.33%), Man (2.39%), and Glc-UA (0.55%) in RFP were all higher than those in the other three varieties (Figure 3D).

#### 3.3.2. FT-IR Characterization

FT-IR spectroscopy is a widely utilized technique for the structural analysis of polysaccharides, providing important information on the functional groups of polysaccharides. As depicted in the results shown in Figure 3C, the FT-IR spectra of the four grape polysaccharides were similar, indicating that polysaccharides extracted from different grape varieties possessed comparable structural characteristics. The intense signal absorption peak in the vicinity of 3400 cm^−1^ for all four grape varieties may be caused by O-H stretching vibration. The absorption band appearing near 1600 cm^−1^ was attributed to the stretching vibration of C-O or C-C, and the absorption peak at 1400 cm^−1^ was considered to be the bending vibration of C-H or O-H, indicating that all the four polysaccharides were acidic. In addition, the absorption between 1000 and 1200 cm^−1^ may correspond to the stretching vibration of C-O-C, indicating the presence of pyranose.

#### 3.3.3. SEM Analysis

The morphological characteristics of polysaccharides were detected by using scanning electron microscopy. As shown in Figure 3E, the surfaces of the four grape polysaccharides showed clear differences. The VFP showed a dense wrinkled texture, and the VMP showed an irregular massive structure. However, the surface of RFP showed a smooth surface with granular protrusion features. The MLP had a rough surface and contained many irregular depressions.

### 3.4. Antioxidant Activity Analysis

Free radicals have been linked to a variety of diseases, including damage to the gastric mucosal barrier. Antioxidants are a class of substances that can remove free radicals from the body or inhibit their production [29]. The antioxidant activities of polysaccharides from different grape varieties were compared by measuring the scavenging rates of DPPH, ABTS, and OH^−^ radicals.

#### 3.4.1. DPPH Radical Scavenging Activity

**Figure 4:**
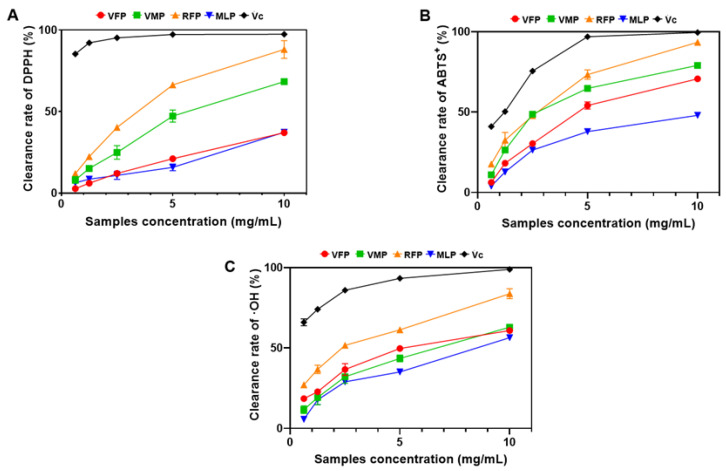
Antioxidant effects of the polysaccharides. (**A**) DPPH radical scavenging capacity, (**B**) ABTS radical scavenging activity, and (**C**) OH^−^ scavenging activity. All studies were performed in triplicate; data are displayed as the means ± standard deviation.

The results in Figure 4A depict the DPPH radical scavenging activity of Vc and the four grape polysaccharides at different concentrations. Within the concentration of 0.625–10.0 mg/mL, the scavenging ability of RFP was higher than that of other grape polysaccharides VFP, VMP, and MLP. DPPH radical scavenging activity was observed to increase with increasing RFP concentration, indicating a dose-dependent increase in scavenging activity. When the amount of addition was 10 mg/mL, the highest inhibition rate of RFP reached 82.25%.

#### 3.4.2. ABTS Radical Scavenging Activity

As shown in Figure 4B, the ABTS radical scavenging ability of all four grape polysaccharides increased significantly with increasing concentration, showing a concentration dependence. When the concentration of RFP was 10 mg/mL, the scavenging ability of RFP was 92.13%, which was the strongest among the four grape polysaccharides. These results indicate that RFP had a stronger ability to scavenge ABTS radicals than VFP, VMP, and MLP.

#### 3.4.3. OH^−^ Radical Scavenging Activity

Figure 4C shows the OH^−^ radical scavenging ability of the four grape polysaccharides. With the increase of polysaccharide concentration, the scavenging effect was enhanced in a dose-dependent manner within the range of 0.625–10.0 mg/mL. At the same concentration, the scavenging ability of Vc was always higher than that of grape polysaccharide. When the concentration reached 10.0 mg/mL, the scavenger activity was as follows: RFP (80.26%) > VMP (61.34%) > VFP (60.17%) > MLP (58.34%).

Based on the determination of the above three antioxidant activities, the RFP showed a stronger antioxidant effect than the other varieties. This may be due to the larger molecular weight of RFP and the higher content of uronic acid. Therefore, we selected RFP for the following animal experiment.

### 3.5. The Effect of RFP on Ethanol-Induced Gastric Mucosal Injury in Rats

**Figure 5:**
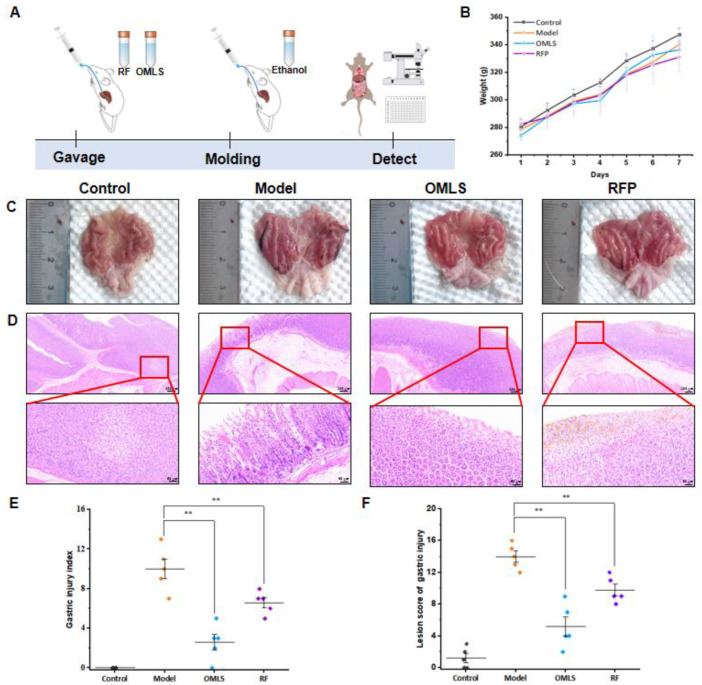
Gastric protective activity of RF on ethanol-induced gastric mucosal injury in rats. (**A**) Schematic diagram of the experimental flow. (**B**) Changes in weight of rats. (**C**) Effects of the degree of gastric mucosal injury in each group. The rats were divided into control group, model group, positive group (OMLS), and RFP group. (**D**) HE staining of gastric tissue after ethanol-induced gastric ulcer. Scale bar was 100 μm and 25 μm. (**E**) Gastric mucosal injury index in rats. (**F**) Microscopic lesion score of gastric mucosae in rats, ** *p* < 0.01, versus model.

In order to evaluate the protective effect of RFP on gastric mucosal injury, rats were given RFP for 7 days and then alcohol to create a gastric mucosal injury model. After the rats were dissected, the gastric mucosal lesions were observed, and the gastric injury index and the gastric mucosal lesion score were calculated. The schematic diagram of the experimental process is shown in Figure 5A. During the experiment, the body weight of all the rats gradually increased, indicating that RFP had no significant effect on the behavioral signs of the mice (Figure 5B).

The gastric tissue observation images of rats in each group are shown in Figure 5C. Compared with the control group, the gastric mucosa injury of the model group was serious and there was obvious bleeding. Compared with the model group, the gastric mucosa injury symptoms of the OMLS group and the RFP group were significantly reduced. According to Figure 5E, it can also be found that the gastric mucosal injury index in the model group was the highest, that in the OMLS group was the lowest, and that in the RFP group was significantly lower than that in the model group (*p* < 0.05), indicating that RFP had a certain protective effect on ethanol-induced gastric mucosal damage.

HE staining results of gastric mucosal tissue samples of rats in each group are shown in Figure 5D. Compared with the model group, gastric mucosal bleeding, necrosis, and inflammatory cell infiltration in OMLS and RFP groups were less severe. The gastric mucosal lesion score in Figure 5F shows that the lesion degree of the model group was the most serious, reaching 14.0 points, and the OMLS group and RFP groups were significantly improved (*p* < 0.05), by 5.2 points and 9.8 points, respectively. This suggests that RFP can promote gastric mucosal repair.

### 3.6. Effects on Biochemical Indexes of RFP on Ethanol-Induced Gastric Mucosal Injury in Rats

**Figure 6:**
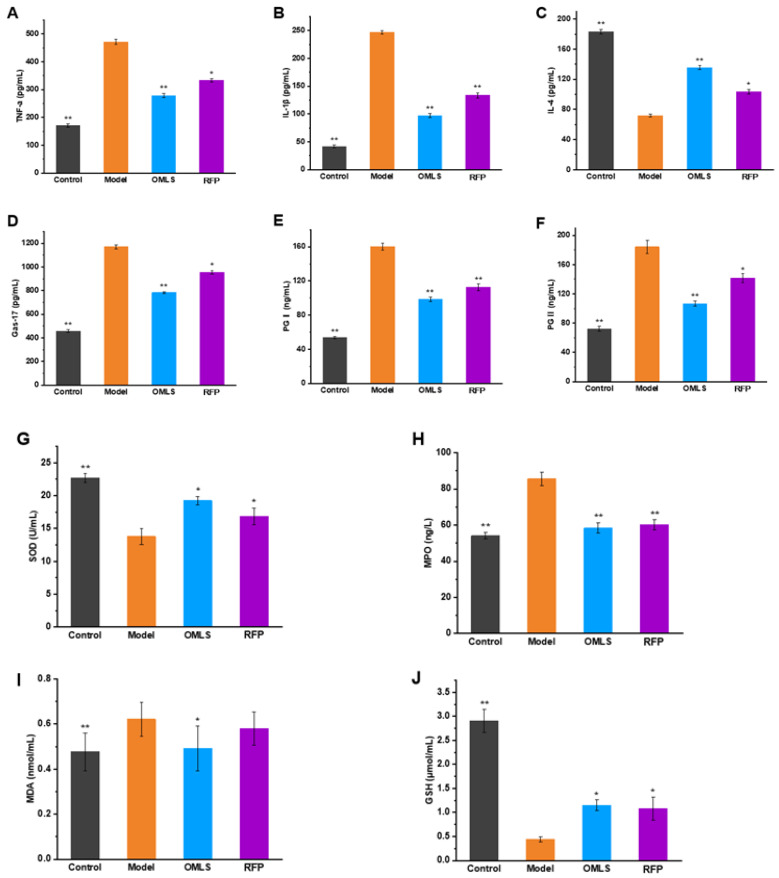
Effects of RFP on serum biochemical indexes of rats in each group. (**A**–**C**) Expression of inflammatory factors in the gastric tissues of rats. (**D**–**F**) Expression of gastric health-related biomarkers of rats. (**G**–**J**) Expression of oxidative stress-related indices in the gastric tissues of rats. * *p* < 0.05, ** *p* < 0.01, versus model.

We used ELISA to detect the effect of RFP on the expression of related inflammatory factors in alcohol-induced gastric mucosal injury in rats (Figure 6A–C). TNF-α and IL-6 are widely considered to be pro-inflammatory factors, while IL-4 is not a typical pro-inflammatory factor: it plays a major anti-inflammatory role in the immune response. We observed that compared with the control group, TNF-α and IL-6 levels were significantly increased and IL-6 levels were significantly decreased in the model group. Compared with the model group, the levels of TNF-α and IL-6 in the RFP group were significantly decreased, and the levels of IL-6 were significantly increased (*p* < 0.05). The results showed that RFP could effectively inhibit the increase of TNF-α and IL-6 levels in serum induced by alcohol, promote the expression of anti-inflammatory factor IL-4, and thus play a protective role against excessive inflammation.

Gas-17, PG-I, and PG-II are all biomarkers closely related to stomach health, and they play an important role in the physiological function of stomach and disease diagnosis. When gastric mucosa is damaged, the number of G cells in antrum may decrease, which affects gastric somatostatin self-regulation, and thus increases gastric mucosa secretion of Gas-17. This elevation may be a compensatory response by the body to repair the damage. Due to inflammation and increased secretion of gastric acid and gastrin, the secretion of PG-I and PG-II is further stimulated. The results showed that the levels of Gas-17, PG-I, and PG-II in the model group were significantly increased (*p* <0.05), while those in the RFP group were significantly decreased (*p* < 0.05). These results suggest that RFP can protect gastric mucosa by inhibiting the excessive secretion of Gas-17, PG-I, and PG-II.

In addition, we determined SOD and MPO activity as well as GSH and MDA levels, which are indicators of mitochondrial oxidative stress (Figure 6G–J). SOD is an important antioxidant enzyme, which can reduce the damage that free radicals do to cells. MPO interacts with H_2_O_2_ to generate reactive oxygen species and active nitrogen molecules, causing oxidative stress and cell damage. MDA is a product of lipid peroxidation and can lead to cell function damage and inflammation. GSH has powerful antioxidant capacity and is able to remove free radicals from the body [30]. In the model group, SOD and GSH levels were significantly reduced compared to the control group, indicating a decrease in antioxidant capacity. MPO and MDA levels were significantly higher than those in the control group, indicating an increase in the degree of oxidative stress. The RFP group significantly increased SOD and GSH levels after gastric mucosal injury, and excessive accumulation of MPO and MDA (*p* < 0.05) was inhibited. These results indicate that RFP can protect gastric mucosa injury by regulating oxidative stress level.

In sum, our study performed an investigation of polysaccharides from four different grape sources. Instead of focusing on commonly studied active components like anthocyanins, we innovatively examined the rarely reported polysaccharide components. Through a series of characterization analyses, we discovered their protective effects against alcoholic gastric mucosal injury. This novel finding contributes to elucidating the biological functions of polysaccharides in grapes and will facilitate the development of grape polysaccharide-based health food products.

Of course, this research has certain limitations at present. The most significant limitation is that we have not yet fully elucidated the structure of the active polysaccharides, or the oligosaccharide sequences that are responsible for the key bioactivities. Therefore, we will continue to conduct in-depth structural analysis and bioactive oligosaccharide sequence studies on the screened grape polysaccharides, thereby laying the groundwork for scaled-up synthesis and establishing a solid foundation for new drug development.

## 4. Conclusions

In this study, we conducted a comprehensive comparison of the chemical composition and structural characteristics of four polysaccharides, and validated the antioxidant activity and gastric mucosal protective effects. Notably, the administration of red-fleshed grape polysaccharide (RFP) effectively suppressed inflammation and free radical generation, offering protective effects against gastric mucosal damage in mice. RFP, with its clearly elucidated structure and demonstrated antioxidation activity, has the potential to become a valuable candidate for development as a gastric mucosal protective drug.

## Figures and Tables

**Table 1:** Box–Behnken experimental design and response values for yield of polysaccharides from grapes.

Run	A: Enzymatic Hydrolysis Time (min)	B: Enzymatic Hydrolysis Temperature (°C)	C: Liquid–Solid Ratio (mL/g)	Yield (%)
1	0 (80)	+1 (65)	+1 (6)	7.28
2	+1 (100)	0 (60)	+1 (6)	6.68
3	+1 (100)	−1 (55)	0 (5)	7.90
4	+1 (100)	0 (60)	−1 (4)	4.54
5	0 (80)	0 (60)	0 (5)	9.09
6	0 (80)	0 (60)	0 (5)	9.26
7	0 (80)	−1 (55)	−1 (4)	7.13
8	−1 (60)	0 (60)	−1 (4)	6.21
9	+1 (100)	+1 (65)	0 (5)	5.83
10	0 (80)	0 (60)	0 (5)	9.04
11	−1 (60)	+1 (65)	0 (5)	7.97
12	0 (80)	−1 (55)	+1 (6)	8.52
13	0 (80)	0 (60)	0 (5)	8.95
14	−1 (60)	0 (60)	+1 (6)	6.98
15	−1 (60)	−1 (55)	0 (5)	8.04
16	0 (80)	+1 (65)	−1 (4)	5.95
17	0 (80)	0 (60)	0 (5)	9.16

**Table 2:** Gastric mucosal injury index.

Injury Degree	1 Point	2 Points	3 Points	4 Points
Blood point	One point for each			
Erosion length	1 mm–5 mm	6 mm–10 mm	11 mm–15 mm	16 mm–20 mm
Erosion Width	1 mm–2 mm	>2 mm		

**Table 3:** Analysis of variance (ANOVA) of the quadratic model (**, significant difference, NS, no significance).

Source	Sum of Squares	df	Mean Square	F Value	*p*-Value Prob > F	Significance
Model	31.17	9	3.46	296.64	<0.0001	**
A—Enzymatic hydrolysis time	2.26	1	2.26	193.39	<0.0001	**
B—Enzymatic hydrolysis temperature	2.60	1	2.60	222.635	<0.0001	**
C—Liquid–solid ratio	3.96	1	3.96	339.37	<0.0001	**
AB	1.0000	1	1.0000	85.65	<0.0001	**
AC	0.4692	1	0.4692	40.19	<0.0001	**
BC	0.0009	1	0.0009	0.0771	0.7893	
A^2^	8.15	1	8.15	698.06	<0.0001	**
B^2^	0.3155	1	0.3155	27.03	0.0013	**
C^2^	10.86	1	10.86	930.48	<0.0001	**
Residual	0.0817	7	0.0117			
Lack of fit	0.0263	3	0.0088	0.6336	0.6313	NS
Pure error	0.1471	4	0.0139			
Cor total	31.25	16				
	R^2^ = 0.9974	Adjusted R^2^ = 0.9940	C.V.% = 1.43			

## Data Availability

The original contributions presented in this study are included in the article/Supplementary Materials. Further inquiries can be directed to the corresponding author.

## Supplementary Materials

The following supporting information can be downloaded at: https://www.mdpi.com/article/10.3390/foods13213500/s1, Figure S1: HPSEC of molecular weight for the standards.

## Abbreviations

BBD: Box–Behnken Design; RSM: response surface methodology; RFP: red-fleshed grape polysaccharide; MAPK: mitogen-activated protein kinase; SOD: superoxide dismutase; GSH-Px: glutathione peroxidase; VF: *Vitis davidii* (Roman. du Caill.) Foex. VM: *Vitis rotundifolia* Michx; RF: red-fleshed grape; ML: Marselan grape; VFP: polysaccharide from VF; VMP: polysaccharide from VM; RFP: polysaccharide from RF; MLP: polysaccharides from ML; TSS: total soluble solids; Mw: molecular weight; HPSEC: high-performance size exclusion chromatography; HPLC: high-performance liquid chromatography.
