# Supplementary figures and images for: Extraction and Structural Characterization of Four Grape Polysaccharides and Their Protective Effects in Alcohol-Induced Gastric Mucosal Injury
Source: Foods. 2024 Oct 31;13(21):3500. doi: 10.3390/foods13213500 (PMC11545244; doi:10.3390/foods13213500)

## Supporting information

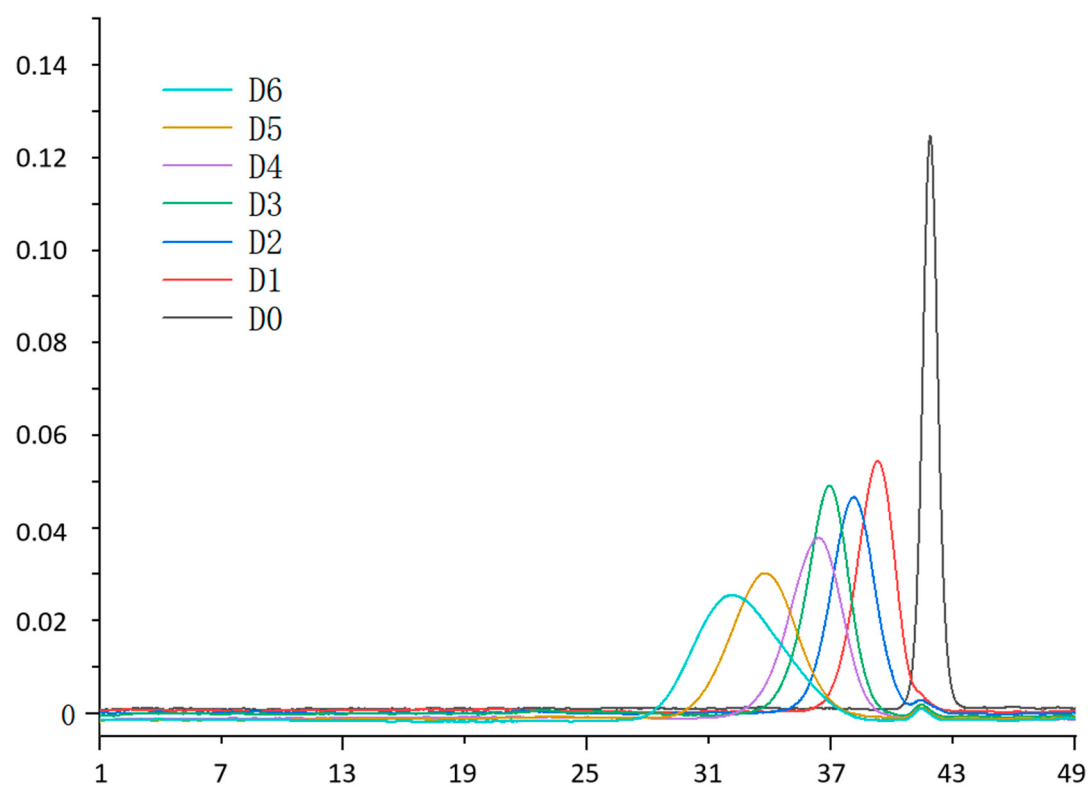

Figure S1 HPSEC of molecular weight for the standards.

Supplement: Supplementary file 1 [file foods-13-03500-s001.zip › foods-3271869-supplementary.pdf]
